# Neuroimaging studies of pediatric social anxiety: paradigms, pitfalls and a new direction for investigating the neural mechanisms

**DOI:** 10.1186/2045-5380-3-14

**Published:** 2013-07-12

**Authors:** Johanna M Jarcho, Ellen Leibenluft, Olga Lydia Walker, Nathan A Fox, Daniel S Pine, Eric E Nelson

**Affiliations:** 1 Section on Developmental and Affective Neuroscience, National Institute of Mental Health, National Institutes of Health, 9000 Rockville Pike, Building 15 K, Bethesda, MD 20892, USA; 2 Section on Bipolar Spectrum Disorders, National Institute of Mental Health, Bethesda, MD 20892, USA; 3 Department of Human Development, University of Maryland, College Park, MD 20742, USA; 4 Department of Quantitative Methodology, University of Maryland, College Park, MD 20742, USA

**Keywords:** fMRI, Development, Peers, Uncertainty, Social cognition, Affect, Behavioral inhibition, Response flexibility, Peer victimization, Bullying

## Abstract

Social Anxiety Disorder (SAD) is a common and debilitating condition that typically manifests in adolescence. Here we describe cognitive factors engaged by brain-imaging tasks, which model the peer-based social interactions that evoke symptoms of SAD. We then present preliminary results from the Virtual School paradigm, a novel peer-based social interaction task. This paradigm is designed to investigate the neural mechanisms mediating individual differences in social response flexibility and in participants’ responses to uncertainty in social contexts. We discuss the utility of this new paradigm for research on brain function and developmental psychopathology.

## Review

### Introduction

Social anxiety disorder (SAD) is a highly prevalent [1] and impairing condition characterized by extreme fear of negative social evaluation and social withdrawal [2]. Because typical SAD onset is in adolescence [3,4], it disrupts normative social behavior during a developmental window critical to maturing peer relationships [5-7]. Moreover, adolescent SAD predicts risk for chronic anxiety and depressive symptoms later in life [8,9]. Despite therapeutic advances, treatment-resistant SAD remains common [10]. Difficulties in identifying novel psychological, cognitive, or brain-based therapeutic targets have impeded progress toward novel prevention and treatment strategies [11]. This difficulty identifying therapeutically relevant targets may reflect the complexity of peer social interactions, a challenge further compounded by the fact that in adolescent SAD, aberrant emotional, cognitive, and behavioral processes emerge during just such interactions. Therefore, neuroimaging paradigms that model real-world peer interactions may help to uncover pathophysiological mechanisms that can then be targeted by novel interventions for SAD.

Indeed, studies demonstrate that when processing social cues, adolescents with, and at risk for, SAD exhibit perturbations between prefrontal and subcortical brain regions, including amygdala and striatum. For example, the anticipation and receipt of one time feedback from numerous unfamiliar peers elicits perturbed fronto-amygdala activity in socially anxious adolescents [12,13], and perturbed fronto-striatal activity in adolescents at high risk for developing social anxiety [14]. However, it is unclear how well existing paradigms map onto day-to-day symptom-eliciting experiences of adolescents with SAD. This calls into question whether fronto-amygdala and fronto-striatal circuits are appropriate targets for novel SAD treatment and intervention strategies. Further, it underscores the need to utilize paradigms with high levels of external validity, which are capable of measuring both brain function and behavior central to adolescent SAD.

Thus, while great strides have been made in understanding the neurobiology of adolescent SAD, methodological challenges have hindered progress towards delineating the neural mechanisms implicated in key features of the disorder. Here we present data from a novel experimental paradigm designed to address some of these challenges. We present these data in the context of a review with four aims: 1) Summarize briefly what is known about biases in SAD that arise when processing social cues; 2) Review results from existing neuroimaging studies that probe social processing in disordered and healthy adolescents; 3) Introduce a novel experimental paradigm designed to assess features of SAD not targeted in existing paradigms; and 4) Propose new directions for research aimed at delineating diagnosis-specific neural circuits that mediate risk for, and expression of, adolescent SAD.

### Cognitive and behavioral biases to social cues in SAD

#### Cognitive biases in SAD

SAD is defined as “a persistent fear of social or performance situations in which the person is exposed to unfamiliar people or to possible scrutiny by others” [2]. This fear is often prospective, such that patients with SAD anticipate that their behavior will result in embarrassment or humiliation during a forthcoming social interaction [2]. Therefore, a key component of SAD is anticipatory worry about social interactions with the real or perceived potential for negative outcomes. This suggests that in contexts with uncertain outcomes, patients with SAD expect adverse social consequences from their behavior, even when the probability for such adverse consequences is low.

Indeed, cognitive theories suggest that biases in perceived self-worth, attention, interpretation, and memory cause individuals with SAD to view social situations through a negatively-distorted lens [15-19]. Such theories suggest that SAD patients have low levels of perceived self-worth, anticipate being held to an unrealistically high performance standard, and expect that failure will result in excessively negative evaluation [16]. Patients with SAD interpret ambiguous social cues as negative or threatening [20-22], and in turn, exhibit an attention bias toward negative or threatening cues (reviewed by [23]). They are more likely to view their performance from an “audience” perspective, focusing on the negative aspects of how they believe they appear to others (e.g., [24-26]). Anxiety generated by this perspective is exacerbated by a heightened vigilance to threat from external cues, such as an audience member rolling their eyes (e.g., [27-29]), and internal cues, like the perception that one is physiologically aroused, blushing, sweating, or shaking (e.g., [30-32]). Finally, patients with SAD are more likely than non-anxious individuals to remember negative social feedback (reviewed by [33]), which is then the subject of subsequent rumination (e.g., [24,25]).

#### Behavioral biases in SAD

The emotional discomfort that results from cognitive processing biases in SAD may shape behavior in unique ways. These behavioral tendencies, in turn, may promote negative social interactions. Two such behaviors are impaired social response flexibility and behavioral disengagement. The complex and rapidly changing nature of social contexts challenges adolescents, regardless of their underlying levels of social anxiety. Competent social behavior requires the ability to adapt flexibly to an ever-changing social milieu. Indeed, response flexibility has been implicated directly or indirectly in a number of models of social competence (e.g., [34-36]). Some suggest that a lack of response flexibility may contribute to poor social competence in patients with SAD [37]. For instance, in social situations, patients with SAD often utilize “safety behaviors,” such as rehearsed or memorized responses (reviewed by [17]). This internal focus of attention may impair detection of contextual factors that influence the behavior of others. Thus, a patient with SAD may assume that an audience member is rolling their eyes to signal disapproval, when in fact the audience member is experiencing discomfort from a contact lens. Patients with SAD are, therefore, unable to adjust their behavior to correspond to the contexts of social interactions. This mismatch between context and behavior is, in turn, associated with negative social interactions [38].

Avoidance or behavioral disengagement in patients with SAD may also be motivated by cognitive biases. A primary symptom of SAD is avoidance of social interactions that provoke anticipatory fear or worry [2]. Such avoidance can be detrimental on many levels; importantly, it prevents patients from experiencing positive social outcomes in feared social contexts, which might otherwise diminish fear [16,38]. Once engaged in a social interaction, cognitive biases related to perceived self-worth, attention, and interpretation may motivate avoidant behaviors such as reduced eye contact, which in turn increase negative social feedback and reinforce existing biases [15,18,39]. Taken together, the interaction of these cognitive and behavioral biases lead to an affective and cognitive structure that is often difficult to treat.

### Neural mechanisms mediating biased processing of social cues in SAD

There are a variety of reasons why defining the neural circuitry associated with SAD may be of benefit to developing approaches to treatment. Typical onset of SAD occurs during adolescence [3,4], a developmental period during which youths transition from family-centered to peer-centered social groups [36]. During this time, significant maturational changes occur in the brain (e.g., [40,41]) and likely contribute to some of the shifts in social behavior observed during adolescence [36]. Delineating the neural mechanisms underlying adolescent SAD may shed light on how normative shifts in social motivation during adolescence contribute to the manifestation of SAD.

Defining the neural mechanisms underlying adolescent SAD may also aid in its nosology. For instance, while the emergence of SAD during adolescence confers an increased risk for chronic psychopathology, many adolescents with SAD overcome their symptoms. Indeed, a majority of adolescents who develop SAD undergo complete remission [9]. Delineating the neural mechanisms of SAD may improve our ability to distinguish among healthy adolescents, adolescents with SAD symptoms that are likely to remit, and adolescents with SAD symptoms that are likely to persist.

Likewise, neuroimaging studies may also help isolate specific forms of aberrant processing among adolescents with SAD that can be targeted by currently available or novel therapies. As noted above, social interactions are highly complex, and rely on processes implicated in perception, interpretation, emotional attribution, and behavioral integration. Identifying neural circuits engaged by social interactions will likely inform our understanding of these aberrant processes and suggest targets for remediation using biological and non- biological methods. Studies have begun to identify the neural circuits mediating biased processing of social cues in individuals with SAD. These studies can be divided into those that use simple or dynamic social stimuli.

#### Simple social stimuli

Relative to healthy adolescents, adolescents with SAD [42-46], or at risk of developing SAD [14,47-51], tend to show heightened amygdala sensitivity and perterbations in striatum and circuits encompassing medial (mPFC) and ventral lateral prefrontal cortex (vlPFC), insula, and anterior cingulate cortex (ACC). Despite the dynamic nature of the cognitive biases that engender symptoms in SAD, many fMRI studies used relatively simple, static stimuli to investigate these perturbations. Because increased reactivity to negative evaluation is a key feature of SAD (reviewed by [17]), such studies often use negative emotional faces as stimuli. Indeed, negative expressions elicit enhanced amygdala response in both adolescents [42,44,46] and adults with SAD ( [42,52-58]; see for alternative findings, relative to healthy individuals [59]). Perturbed activity is also observed in frontal regions connected with the amygdala and striatum, including insula [54,60], ACC [42,61], vlPFC, and mPFC [53,62]. Thus, SAD is characterized by perturbed engagement of a neural network critical for interpreting social cues and regulating or inhibiting affective responses to those cues reviewed by [63].

While these results further our understanding of the mechanisms that support SAD, two factors limit their utility. First, these patterns of brain activation lack disorder-related specificity. Longitudinal, cross-sectional, family-based, and treatment studies find at least some degree of evidence for disorder specificity in SAD relative to other forms of anxiety, such as general anxiety disorder [64,65] panic disorder [64], post traumatic stress disorder [66], and specific phobia [9,66]. Yet, perturbed activity in fronto-amygdala circuits in response to negative emotional faces is not unique to adolescent SAD; it also occurs in adolescents with generalized anxiety disorder [67-69] or other psychiatric conditions, including post traumatic stress [70], bipolar disorder [71], major depressive disorder [72], ADHD [73], and severe mood dysregulation [73]. This may reflect shared emotional disruptions across multiple forms of psychopathology. However, such findings do little to inform SAD-specific diagnostic or treatment options. Thus, further work is needed to delineate the specific neural mechanisms of SAD.

Second, in these studies, static stimuli are presented without a meaningful social context. Because of this, traditional emotional face-processing paradigms do little to illuminate the neural mechanisms associated with perceptual, affective, and cognitive systems that evoke biases in SAD during actual social interactions. To address this issue, neuroimaging studies have begun to utilize more dynamic paradigms designed to engage psychological processes that resemble actual interpersonal interactions.

#### Dynamic social stimuli

In socially dynamic paradigms, participants are led to believe they are being evaluated by, or are receiving real social feedback from peers. This is an important departure from earlier studies because it puts the participant in the social spotlight, a primary concern for patients with SAD. Here, we briefly describe methodology and data from existing dynamic paradigms most relevant to adolescent SAD.

##### *Social*-*evaluative stress*

Some of the earliest neuroimaging studies to implement dynamic social stimuli utilized methods adapted from the Trier Social Stress Test [74]. These paradigms expose participants to social situations that provoke social-evaluative threat, whereby a participant’s performance on a self-relevant task can be judged negatively by others [75]. In such studies, participants are typically required to deliver an impromptu speech to a highly salient but non-responsive audience, or to perform a difficult arithmetic task while an experimenter urges them to ‘go faster,’ and makes their errors highly salient. This elicits high levels of social stress, as indexed by self-report and elevated cortisol response [75].

Although some evidence suggests that social-evaluative threat differentially engages the brain in adults with and without SAD, results are inconsistent. For example, an fMRI study demonstrated that while anticipating delivery of a speech, adults with, relative to without, SAD exhibit heightened activity in amygdala, insula, and striatum, but diminished activity in ACC, mPFC, and dorsolateral PFC (dlPFC) [76]. Yet, when measuring electrical brain activity with electroencephalogram (EEG), anticipated public speaking is associated with heightened activity in dlPFC among adults with, relative to without, SAD [77]. Positron emission tomography (^15^O-H_2_O PET) has been used to examine regional cerebral blood flow (rCBF) during the actual delivery of a speech. These studies find adults with, relative to without, SAD exhibit greater rCBF in amygdala, but less rCBF in both insula and vlPFC [78]. Additionally, SAD adults who respond to pharmacotherapy or cognitive behavioral therapy exhibit decreased pre- to post-treatment amygdala rCBF during a speech [79]. However, a different pattern of rCBF in amygdala emerges with the challenging arithmetic task. Here, adults with and without SAD exhibit *diminished* rCBF in amygdala [80]. Moreover, response to pharmacotherapy is unrelated to changes in amygdala rCBF from pre- to post-treatment [80].

Together, these data suggest that social-evaluative threat in adults with SAD is associated with a complex pattern of perturbed fronto-amygdala and striatal reactivity. The inconsistency in findings across studies could relate to variability in imaging modality (i.e., EEG, fMRI, PET), the temporal proximity of threat (i.e., anticipated or current evaluation), and the self-relevance of the task (i.e., speech or arithmetic). Given the shifts in social, cognitive, and self-related processes that occur in adolescence [36], these latter factors may differentially influence the way that adults and adolescents with SAD engage the brain, and thus experience, self-evaluative threat.

#### Social acceptance/rejection

Paradigms that model social acceptance/rejection typically involve one-time social feedback from numerous unknown peers. In the Chatroom task [12-14,43,81], participants review photos of smiling, unfamiliar peers, and are asked to indicate who they want to chat with online at a subsequent session. Importantly, participants are told that these peers will make the same decisions about them. During fMRI scanning at the subsequent session, participants first predict how interested each peer was in chatting with them, a measure of negative biases about their self-worth. Next, they receive the peer’s acceptance or rejection feedback, which engages processes related to whether the participants accurately predicted the peer feedback.

The Social Judgment paradigm, developed by Sommerville and colleagues [82], resembles the Chatroom task. Prior to imaging, participants review photographs of unfamiliar, smiling, age-matched peers and decide whether they would like the depicted individual. As in the Chatroom task, participants are led to believe the depicted peers will also judge them. During a subsequent fMRI scan, each picture is displayed while participants predict the rating they received from the peer and are then shown the “actual” rating. Finally, in a similar paradigm developed by Davey and colleagues [83,84], participants view photographs of peers and rate how much they think each peer would like them. While undergoing an fMRI scan, participants view each peer and receive either positive or no social feedback.

Although participants engage in self-assessment and receive peer feedback in each of these three tasks, there are important differences. In the Chatroom task, participants believe that they will chat with one of the depicted individuals, while in the other tasks they do not believe an interaction will occur. Second, participants in the Chatroom task predict peer interest in a first run and receive peer feedback in a second run. In other tasks, prediction and feedback for each peer occurs before the presentation of a subsequent peer. While these variations are not dramatic, they may impact results. Nevertheless, because all three paradigms engage participants in a dynamic social context, their external validity is greater than static expression tasks. Finally, it is important to note that only the Chatroom task has been used to study clinically anxious adolescents [43].

Emerging data from studies utilizing these paradigms suggest that discrete social contexts engage neural mechanisms closely linked with behavioral responses characteristic of SAD. For example, anxious adolescents who perform the Chatroom task demonstrate the expected bias in perceived self-worth, reporting that their peers will be less interested in chatting with them than healthy individuals [43]. When predicting feedback from rejected peers, anxious adolescents demonstrate greater amygdala and vlPFC activity relative to non-anxious adolescents [43]. Prior to receiving peer feedback, amygdala activity is heightened in both anxious and healthy adolescents; after receiving feedback, this activity declines in healthy but not anxious participants [12]. Adolescents with high levels of stable childhood behavioral inhibition, a temperament that increases risk for SAD, exhibit perturbed striatal engagement when predicting or receiving peer feedback [14]. In the Social Judgment task, adults with low self-esteem, a correlate of SAD and other forms of psychopathology, exhibit heightened mPFC activity to acceptance relative to rejection feedback, and subsequently recall fewer instances of peer acceptance [85]. Finally, in a study of young adults, depressed patients with high rates of comorbid anxiety exhibit heightened amygdala response to acceptance feedback relative to controls [84]. Because most of these studies include relatively small, heterogeneous populations, the results should be considered preliminary. Nevertheless, these findings implicate perturbed fronto-amygdala and fronto-striatal circuits during the anticipation of, and response to, social contexts in adolescents with features of SAD.

#### Social inclusion/exclusion

Unlike paradigms that model single instances of social acceptance/rejection from unfamiliar peers, social inclusion/exclusion can be modeled with repeated real-time interactions with two peers. In this model, a “relationship” is established between the participant and their peers across repeated interactions. To date, two such tasks have been developed: the Cyberball task and the Chat-Interact paradigm. In the Cyberball task, participants engage in a virtual ball tossing game with two unfamiliar peers represented by cartoon figures [86]. Photographs of age and gender-matched peers sometimes accompany these figures [87]. The manipulation consists of instances where the research participant is systematically included or excluded from the game. The Chat-Interact task models high or low levels of social exclusion from topical discussions by peers who purportedly know the participant’s interests [88]. One peer disproportionately includes the participant, and the other disproportionately excludes the participant from discussions.

Data from both tasks suggest that repeated exclusion is highly salient in psychiatrically healthy populations. In the Cyberball task, both adolescents and adults report greater anxiety or distress during exclusion relative to inclusion [87,89,90], whereas social exclusion from the Chat-Interact task results in pupil dilation, an index of arousal [88]. fMRI studies with Cyberball in adolescents report that heightened insula and subgenual ACC activity are related to higher levels of distress during exclusion, an effect that may be regulated by vlPFC [89,90]. Moreover, healthy adolescents with greater activity in subgenual ACC during exclusion, relative to inclusion, are more likely than adolescents with lower activity on the same contrast to exhibit subclinical depressive symptoms one year later [91]. Neuroimaging findings with the Chat-Interact task are still preliminary but bear some resemblance to both Cyberball and social acceptance/rejection tasks described above. Specifically, during exclusion, depressed, relative to non-depressed adolescents, exhibit increased amygdala, insula, and subgenual ACC activity [92].

#### Limitations

Great strides have been made in modeling adolescent peer interactions during functional neuroimaging. While still relatively new, existing paradigms hold promise for elucidating the neural and psychological underpinnings of SAD. Despite these advances, some key features of SAD are not modeled by existing paradigms. For example, social-evaluative threat paradigms typically involve performing in front of a non-responsive audience or interfacing with a confrontational authority figure. Neither situation engages key aspects of day-to-day social interactions with peers, events highly salient for the adolescent. Further, while behavior is a key component of social-evaluative threat paradigms, coding of such behavior is typically done through video tape recording and observer ratings (e.g., [93]), methods that are not appropriate in the scanning environment. Available acceptance/rejection paradigms rely on categorical responses that require participants to make dichotomous choices about unfamiliar peers, who in turn provide one-time positive or negative social feedback. Inclusion/exclusion paradigms include repeated interactions, thus avoiding some of these shortcomings, but they suffer from others. For example, the Chat-Interact and Cyberball paradigms do not acquire behavioral responses to inclusion/exclusion. Such responses are needed to assess associations between brain function and cognitive or behavioral biases in social processing linked with SAD. Moreover, both paradigms typically rely on block designs, and thus are not optimized to assess brain function in discrete phases associated with the anticipation, and subsequent receipt, of social feedback.

Two other key elements are lacking in existing imaging paradigms that model social interaction. The first is uncertainty. Symptoms of SAD are precipitated by the possibility that future social situations may result in negative social feedback, regardless of the probability of such an outcome [2]. As a consequence, patients with SAD often respond to uncertain social situations with distress or avoidance. Although uncertainty about social feedback is a core symptom-eliciting context for SAD, it is not manipulated directly by any current social-interaction paradigm. Studies using the Chatroom paradigm demonstrate that, relative to healthy and low-risk peers, adolescents with, or at high risk for, SAD differentially engage fronto-amygdala-striatal circuitry while they anticipate feedback [43] or receive negative feedback [12]. However, it is unclear if these differences are driven by the anticipation and receipt of social feedback generally, or of uncertain social feedback specifically. A paradigm that systematically manipulates level of social uncertainty would therefore fill an unmet need in SAD research.

The second key element is response flexibility to social contexts. Behavioral disengagement and inflexibility are two important symptoms of SAD that can manifest in patterns of rehearsed responding or avoidance of social engagement altogether. In existing paradigms, participants respond to social situations with either open-ended behavior that is difficult to capture during imaging (e.g., social-evaluative threat), or using dichotomous responses. For example, participants select or reject peers in Chatroom, Chat-Interact, and Social Interaction tasks, and toss a virtual ball to one or another peer in Cyberball tasks. Thus, existing social interaction tasks cannot model the relationship between brain function and variability in behavioral response, a key element of SAD. Response flexibility in adolescents with and without SAD may vary depending on the degree of uncertainty associated with peer feedback. While many aspects of social competence have already been established in adolescence, refinements and alterations in neural circuits that promote successful social functioning continue well beyond puberty [36,37,94]. Perturbations in these circuits, and corresponding disruption in behavior, may be more evident in uncertain or ambiguous, rather than clear-cut social situations in which appropriate responses are more apparent.

Finally, existing paradigms are designed to study individuals during late adolescence and young adulthood – the time when SAD typically first manifests. However, many of the psychosocial biases that mediate SAD likely precede the onset of full-blown symptoms. Therefore a paradigm designed specifically to study social interactions in pre-pubertal juveniles may be particularly beneficial.

### A novel approach: the virtual school paradigm

The “Virtual School” paradigm was developed to address these limitations by modeling anticipation to, and social feedback from, positive, negative, or unpredictable peers, to whom participants provide psychologically meaningful behavioral responses. Using a school-like context as a backdrop, these purported peers are represented by cartoon avatars, each of which has a reputation for being nice, mean, or unpredictable. Below, we describe methods and data from a preliminary behavioral study used to develop the fMRI paradigm.

#### Methods

##### Participants

Fifteen children (10.25 ± 1.37 years; 9 female) were recruited from the community and studied at National Institute of Health or University of Maryland. Informed consent/assent was obtained from all parents/participants; University of Maryland, College Park Institutional Review Board and the Combined NeuroScience Institutional Review Board at the National Institute of Health.

#### Procedure

At an initial visit, participants are told they will play a game called Virtual School. At the Virtual School, the participant learns they will be the “New Kid,” and that they will interact with “Other Students,” who are purportedly peers that previously played the game. Participants complete a personal profile, consisting of a name to be used on-line, answers to multiple-choice questions about their preferences (e.g., movies, music), and answers to two open-ended questions that let the Other Students know more about them (programmed with TCL). Participants then create a cartoon avatar (My Avatar Editor), and are told that the Other Students will review their profile and avatar prior to their next visit. As with existing dynamic social paradigms, to maintain experimental control, participants do not actually interact with peers, but with a computer program that has pre-determined the characteristics of the Other Students.

#### Pre-social interaction procedures

**Figure 1:**
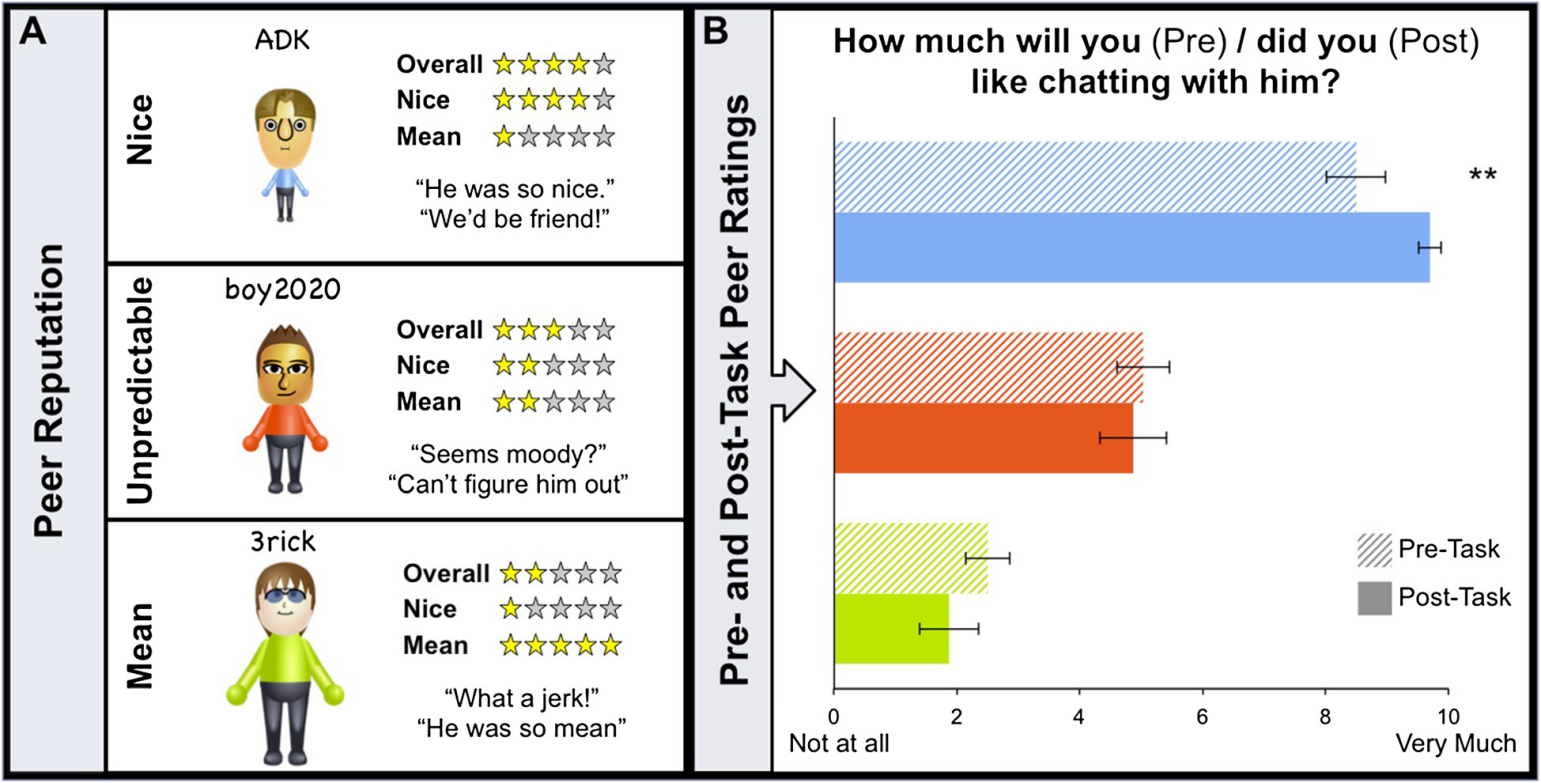
**Example of nice, unpredictable, and mean peers (A), with corresponding pre- and post-social interaction interest in peers (B).** Prior to completing the task, participants are shown the Other Students’ avatars, and on-line names. Ratings and comments, purportedly provided by previous New Kids, indicate the Other Students have a reputation for being nice, unpredictable, or mean **(A)**. To test how these reputations influence interest in peers, participants are asked to rate how much they *will* (pre-task; hatched bars) or *did* (post-task; solid bars) like chatting with each peer **(B)**. Blue = Nice; Red = Unpredictable; Green = Mean. **p < .005.

Approximately two weeks after the initial visit, participants return to attend the Virtual School. Prior to actually engaging with the Other Students, participants are shown Other Students’ avatars, on-line names, along with ratings and comments about the Other Students, purportedly provided by previous New Kids (Figure 1A). Participants learn that two of the Other Students have a reputation for being ‘Nice’, two for being ‘Mean’, and two for being ‘Unpredictable.’ Thus, Nice and Mean peers respectively model predictable positive and negative social evaluation outcomes, while Unpredictable peers model uncertain social evaluation outcomes. Reputations are revealed prior to attending the Virtual School to minimize variability of participant learning during social interactions, establish discrete socio-emotional expectations for each peer, and to model real world social contexts, which often include interacting with peers with distinct and known personalities. To eliminate potential confounds associated with physical attributes, peer reputations are randomly assigned to the 6 gender-matched avatars across participants.

To determine how well participants learn these reputations, children then rate the personality of each Other Student on a ten point scale, in which 0 indicates the peer is mean, 10 indicates the peer is nice, and 5 indicates the participant is uncertain about the peer’s personality (e.g., “can’t tell”). Additionally, to determine the extent to which reputation influences expected interest in interacting with peers, participants then rate how much they think they will like chatting with each Other Student on a 0 (not at all) to 10 (very much) scale.

#### Social interaction at the virtual school

Although this preliminary study was completed outside of an fMRI scanner, procedures for this portion of the task were optimized for fMRI-based data collection and analysis. As such, fMRI-based terminology will be used to describe this portion of the methods. The social interaction task is completed across 4, 9-min runs. To minimize fatigue, each run includes 2 blocks (one classroom per block), separated by a brief rest. All 6 Other Students appear in each classroom, and are randomly assigned to seats in each room. The New Kid’s visual perspective is from the front of the room, where they can see all of the Other Students.

**Figure 2:**
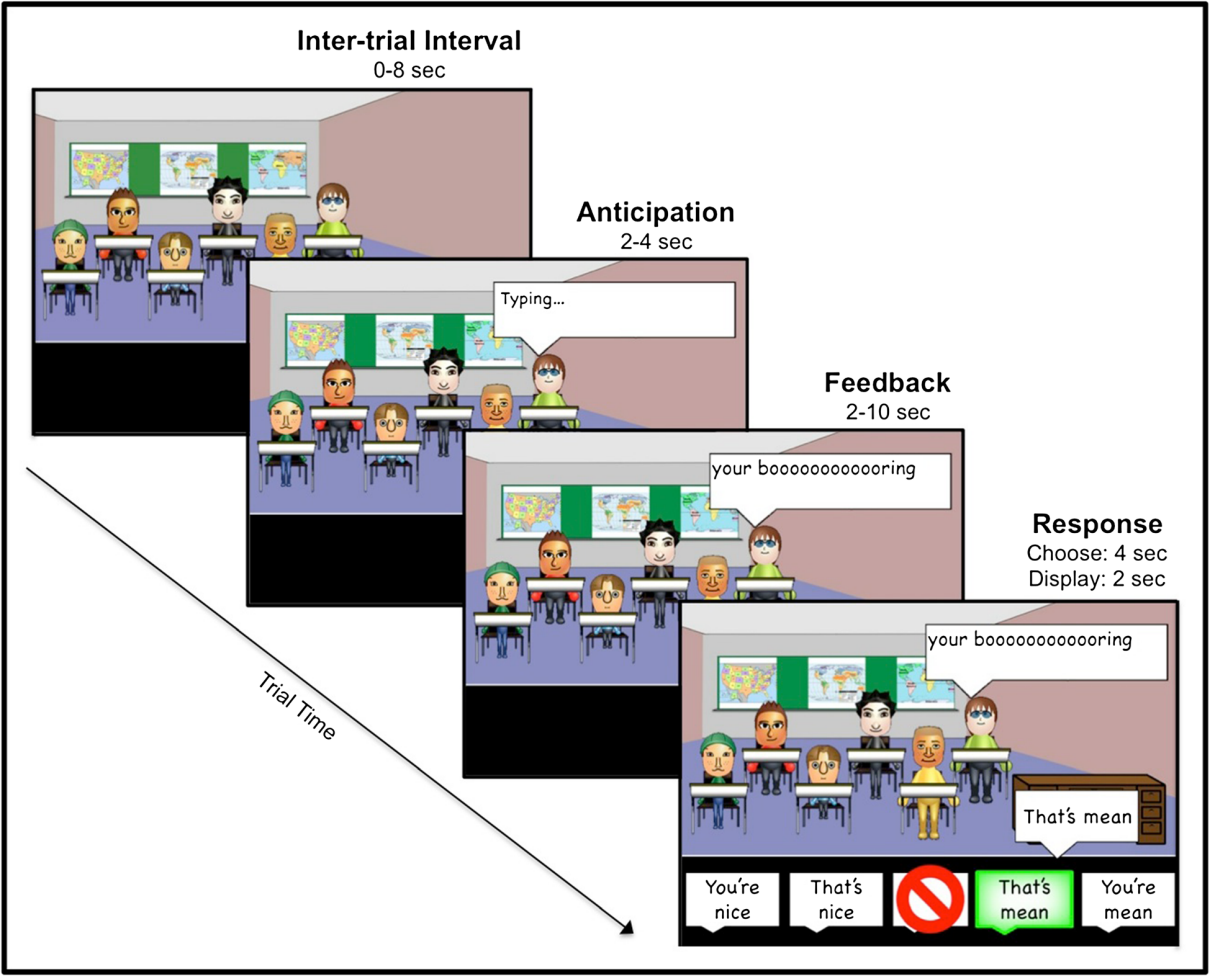
Timeline of a trial.

Each run includes 24 trials separated by an inter-trial interval (0–8 sec; M = 4 sec). Each trial (see Figure 2) begins when the word “Typing…” appears above one of the Other Students (2–4 sec; M = 3 sec). This is followed by a written comment directed at the New Kid/Participant (2–10 sec; M = 6 sec). These comments are either positive (e.g., “Cool avatar!”) or negative (e.g., “You’re lame.”). To strengthen the perception that they are interacting with real peers, half of the comments reference information from the participant’s personal profile (e.g., “You like Justin Bieber? You’re lame.”). Nice and Mean peers provide 100% positive or negative social feedback. Unpredictable peers provide 50% positive and negative social feedback.

Participants then use a button box to respond with one of 5 options (4 sec). As depicted in Figure 2, all 5 options appear at the bottom of the screen. There are two positive response options (You’re nice, That’s nice), two negative response options (You’re mean, That’s mean), and an avoidant response option (∅). Participants are told that they must choose an option each time they receive feedback; choosing the avoidant option means that they can forgo providing a positive or negative response and “ignore” the feedback. Thus, participants can disengage from Other Students, while still providing behavioral data that can be coded as avoidant.

The opportunity to respond establishes an interactive context that models the way anxious adolescents respond to real-world positive or negative social feedback. Participants are allowed the flexibility to direct their positive and negative responses toward the content of the feedback they received (i.e., “That’s nice” or “That’s mean”), or toward the peer who delivered the feedback (i.e., “You’re nice” or “You're mean”). This flexibility may provide insight into whether participants attribute social feedback to situational factors (i.e., “That’s nice” or “That’s mean”), or to the intrinsic nature of their peers (i.e., “You’re nice” or “You're mean”). Responses are then displayed on the screen, or omitted in the case of avoidant responses (2 sec).

Thus, each trial includes the following 3 types of events: 1) anticipated social evaluation from Nice, Mean, and Unpredictable peers; 2) receipt of social evaluation, which includes positive feedback from Nice and Unpredictable peers, and negative feedback from Mean and Unpredictable peers; and 3) participant response to social evaluation. In all there are 28 trials for each reputation type.

#### Post-social interaction procedures

After completing their social interactions at the Virtual School, participants report how much they actually liked chatting with each of the Other Students. They are then given a chance to write open-ended comments that they believe will be shared with the next New Kid, thus providing information that purportedly contributes to the reputation of the Other Students. Deception is assessed prior to debriefing participants.

#### Data analysis and results

Behavioral responses were averaged across each pair of Other Students embodying each type of reputation (Nice, Mean, Unpredictable). To determine how well participants learned peer reputations prior to engaging in social interactions at the Virtual School, a repeated measure analysis of variance (ANOVA) was performed on personality ratings participants provided for Other Students with each type of reputation (Nice, Mean, Unpredictable). There was a main effect of reputation on the personality ratings (*F*(2,13) = 34.27, *p* < .001). Specifically, Nice peers (M ± SD, 8.60 ± 1.53) were rated more highly (i.e., “nicer”) than Unpredictable peers (4.5 ± 1.38), who were in turn rated more highly than Mean peers (2.2 ± 1.75). Contrasts were statistically significant (*p* < .001) for each pair-wise comparison. This indicates that children learned the reputation of peers prior to initiating social interactions in the Virtual School.

Next, a 3 (Reputation: Nice, Mean, Unpredictable) X 2 (Time: Pre-task, Post-task) repeated measures ANOVA was performed to determine if participant interest in chatting with the Other Students changed from pre- to post-social interaction at the Virtual School, depending on peer reputation. A Reputation X Time interaction emerged for ratings of interest in chatting with peers (*F*(2,13) = 5.04, *p* < .02; Figure 1B). This was driven by a significant increase in “interest-in-chatting” ratings from pre- to post-social interaction for Nice peers (*t*(14) = 3.90, *p* < .005), but no change in ratings for Unpredictable or Mean peers. Thus, first-hand social interactions with Nice peers augment the positive impressions healthy children formed using second-hand information about peer reputation. It will be informative to determine if children with, or at risk for, SAD exhibit the same pattern of augmented impressions for Nice peers only, or if their impressions of Mean and Unpredictable peers will also be augmented by first hand interactions.

**Figure 3:**
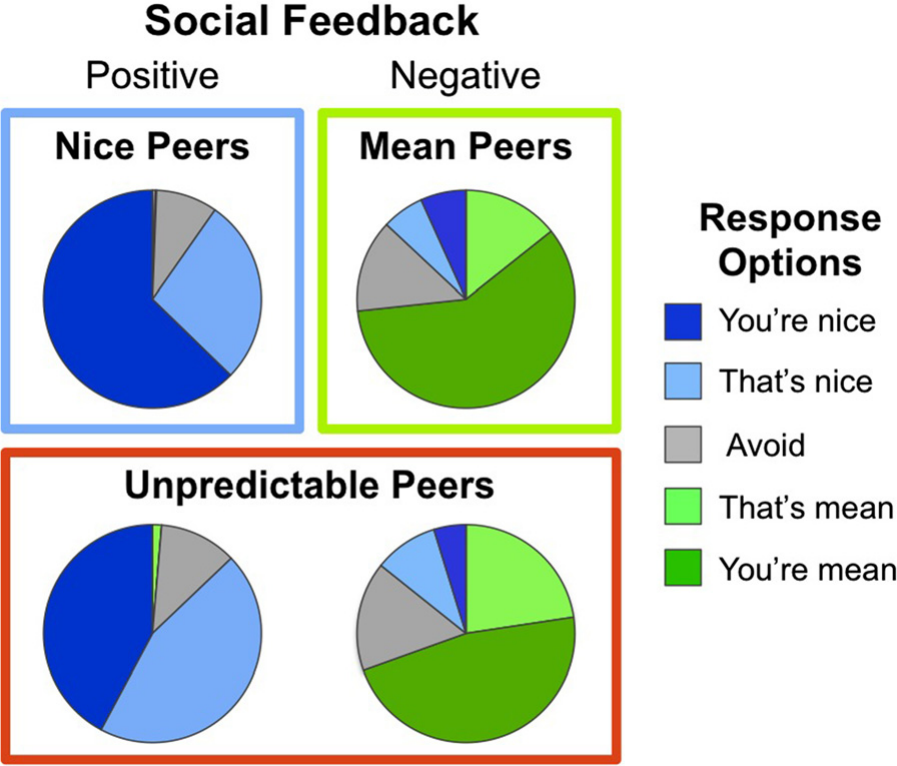
**Response frequency to positive and negative social feedback from nice, mean, and unpredictable peers.**

Finally, analyses were conducted on participant behavioral responses during social interactions at the Virtual School. This was done to determine if frequency of response type varied based on both the valence of the comments they received (positive, negative) and the reputation of the peers who delivered them (Nice, Mean, Unpredictable). Specifically, a 5 X 4 repeated measures ANOVA was performed to determine if the frequency with which participants utilized each of the 5 response options varied based on the 4 types of social feedback they received (positive comments from Nice peers, positive comments from Unpredictable peers, negative comments from Mean peers, negative comments from Unpredictable peers). Analyses of participant response frequency to social evaluation revealed a robust Response Frequency X Social Feedback interaction (*F*(1,12) = 22.17, *p* < .001), suggesting that healthy adolescents make different (flexible) responses to their peers, depending on peer reputation and valence of the comments received (Figure 3).

To determine what was driving the Response Frequency X Social Feedback interaction, 3 sets of planned pair-wise comparisons were conducted using paired sample t-tests. The first analysis assessed response frequency to positive comments compared with negative comments. Regardless of reputation, positive comments yielded more positive responses (“That’s nice” or “You’re nice”) than negative responses (“That’s mean” or “You’re mean”), and vice versa (both analyses yielded *t*(14) > 9.00, *p* < .001). The second analysis assessed response frequency to positive comments from Nice peers compared with positive comments from Unpredictable peers. Participants were more likely to respond with “You’re nice” to positive comments from Nice peers compared with positive comments from Unpredictable peers *t*(14) = 3.05, *p* < .01. The final analysis assessed response frequency to negative comments from Mean peers compared with negative comments from Unpredictable peers. No significant differences emerged. Frequency of avoidant responses (∅) did not vary in any of the 3 sets of planned pair-wise comparisons. Finally, a small, but notable number of positive responses were provided following negative comments from Mean and Unpredictable peers. During debriefing, participants reported using this response pattern to express sarcasm.

All 15 participants reported being deceived, which was supported by the open ended responses participants provided about the Other Students for purported future New Kids (e.g., Nice peer: “*He is and was truly nice at heart*.”; Unpredictable peer: “*He was ok*- *trying to fit in with the bullies but sometimes cool*.”; and Mean peer: “*He was not very nice and I never want to talk with him again*.”).

#### Novel contributions of the virtual school paradigm

Together, these data suggest that the social contexts modeled by the Virtual School paradigm engage discrete psychological processes, indexed by both self-report measures and variability in response selection. When used in conjunction with fMRI, we believe the Virtual School paradigm will make 4 novel contributions to our understanding of adolescent SAD. First, unlike existing paradigms, the Virtual School paradigm can explicitly contrast brain activity engaged during the anticipation and receipt of uncertain or predictable social feedback, modeled by Unpredictable peers, and Nice or Mean peers, respectively. Given that the anticipation of unpredictable social outcomes is a particularly potent means for eliciting fear or worry in patients with SAD, this aspect of the paradigm may shed new light on neural circuits engaged by such contexts. Second, the Virtual School paradigm can explicitly contrast brain activity engaged during the anticipation and receipt of predictably positive or negative social feedback, modeled by Nice and Mean peers, respectively. Although prior studies demonstrate that healthy and SAD adolescents differ in fronto-striatal activity while anticipating predictable monetary gain or loss [45], parallel work has not been done in the social domain. Third, unlike existing social neuroimaging tasks, the Virtual School paradigm provides participants with the opportunity to make flexible behavioral responses to social feedback from different types of peers. Thus, the Virtual School paradigm can clarify the neural mechanisms associated with blunted response flexibility to social cues, a key feature of SAD e.g., [34-36]. Finally, the Virtual School paradigm can model avoidant responding, another key feature of SAD not assessed by existing paradigms. However, it must be noted that, while participants have the ability to respond flexibly to social feedback, the number of possible responses is limited. Therefore, participants who find the available responses unsatisfactory may choose an avoidant response. These novel contributions of the Virtual School paradigm could advance research on the neural mechanisms underlying adolescent SAD, thus facilitating the design of prevention or treatment interventions.

### Future directions

Once implemented with fMRI, we expect the novel design features of the Virtual School paradigm to provide important insights into the neural circuit perturbations and cognitive biases that characterize adolescent SAD. Moreover, because it utilizes school-like contexts that are highly relevant during childhood [95-98], the Virtual School paradigm may facilitate the study of neural circuits engaged by social contexts in young children, particularly those at heightened risk for SAD.

#### Children at risk for SAD

A number of risk factors have been implicated in heightened risk for SAD, including individual differences in genetics (e.g., [99,100]) and temperament [101,102], and in environmental factors, such as parenting style and exposure to stress or peer victimization [103]. Here we provide examples to demonstrate the utility of the Virtual School paradigm for assessing the neural correlates of risk factors that emerge at developmentally distinct periods: behaviorally inhibited temperament (BI), which manifests during infancy [104], and peer victimization or bullying, which typically occurs in childhood [105].

#### Early-emerging risk: behaviorally inhibited temperament

BI is a temperament identified in approximately 10–15% of infants [106], which manifests as heightened reactivity to novelty, persistent childhood social reticence, and chronic fear of social rejection [104,107]. Adolescents with BI have a difficult time forming friendships [107] and are 4–7 times more likely to develop SAD than their non-inhibited peers [101,102]. Stable childhood BI also predicts a pattern of fronto-amygdala and fronto-striatal perturbation in adolescence and adulthood that resembles perturbations observed in SAD [14,43,47-51]. Indeed, we recently found that, compared to adults with no history of BI, young adults with stable childhood BI exhibit a blunted ventral striatal response to positive social feedback in the Chatroom task [14]. This suggests one long-term consequence of BI may be a diminished appetitive response to social reward. BI appears in infancy, and is likely moderated by cognitive and contextual factors across maturation (reviewed by [104,108]). Thus, investigating the neurocognitive mechanisms engaged by social contexts in young children with BI may be particularly fruitful. The Virtual School paradigm is tailored for just such research.

#### Later-emerging risk: childhood bullying

Peer victimization is most prevalent during childhood and adolescence [105], when peer acceptance is most salient [5,109] and onset of SAD is most common [3]. Over 30% of children experience peer victimization or cyber-bullying [110], which often results in long lasting negative outcomes, including SAD [64,111-113]. Despite its pervasiveness, little is known about the mechanisms by which peer victimization confers risk for SAD, or the factors that facilitate resilience among victims. Indeed, the relations between current peer victimization and neurocognitive perturbations in social contexts are unknown. The Virtual School paradigm includes social contexts that may be particularly salient for victimized adolescents. Previous experience with bullying or other forms of social aggression may sensitize neural circuits to negative social encounters and precipitate behavioral patterns that increase the risk of future bullying and SAD. In addition, the Virtual School paradigm conforms to some of the elements of bullying, and thus may provide insight into this type of interaction. Specifically, the paradigm involves repeated or on-going negative social feedback delivered by peers who could be considered higher status than the participant (i.e., the peers are existing students who can generate unique comments based on the participant’s profile). Moreover, given the ubiquity of Internet exposure, the proliferation of on-line social networks and cyber-bullying (e.g., [114,115]), the Virtual School paradigm can address the special need of determining the effects of victimization in a culturally relevant “virtual” social context.

#### Potential for SAD interventions

Traditional treatment options for adolescent SAD include pharmacotherapy, cognitive behavioral therapy, and interpersonal therapy. Although adults with SAD have high response rates with these treatments (50–85%), treatment resistant symptoms and remission are still too common (33–75%) [10]. By implementing the Virtual School paradigm in adolescents with and without SAD, as well as in non-anxious adolescents at high and low risk for SAD, the neural circuits associated with risk for, and resilience against, psychopathology may be identified. As such, we expect that the Virtual School paradigm will facilitate progress towards developing empirically driven, novel interventions to target perturbed neural circuits.

With some modifications, the Virtual School paradigm could be used as a tool to train at-risk and affected adolescents to engage neural circuits associated with resilience during social interactions. Indeed, interventions developed based on findings from translational neuroscience have shown promise in the treatment of anxiety. For example, anxious patients tend to exhibit attention biases toward threatening static social stimuli [23,116], a bias linked with fronto-amygdala dysregulation in anxious adolescents and adults [67,68,117]. Attention Bias Modification Therapy (ABMT) trains anxious patients to eliminate this bias and results in reduced anxiety symptoms [118-120]. This novel and promising treatment is thought to function by normalizing fronto-amygdala response to threat [121]. We hypothesize that while attending the Virtual School, adolescents with SAD will exhibit diminished response flexibility to social feedback and corresponding perturbation in brain function. If this is the case, then adolescents with, or at risk for, SAD could be trained to respond, or attend to feedback, in a more flexible way. Like ABMT, such training may normalize brain function, thereby reducing social anxiety. This training could be done implicitly, as in ABMT, or explicitly via coaching.

The Virtual School paradigm and similar experimental models may provide researchers and clinicians a unique opportunity to isolate and treat aberrant processes engendered by specific aspects of complex social interactions. For instance, treatment or coaching could focus on dysregulated responses that occur during the anticipation of uncertain social evaluation or during response selection to positive or negative social evaluation. Moreover, the Virtual School may allow clinicians to perform exposure therapy or biofeedback training in a realistic social context that can be tightly controlled and uniquely tailored to patient needs. Although a great deal of work would need to be done before implementing such interventions, one can speculate that such strategies would be plausible.

#### Beyond SAD

The importance of social interactions increases during adolescence [5-7]. Therefore, studies that use the Virtual School paradigm in youth ranging in age from early childhood to late adolescence would inform our basic understanding of normative developmental changes in the neural circuits engaged by social interactions. Additionally, with minor modifications, this paradigm could be used to assess the neural correlates of expectancy bias, stereotypes, and context conditioning. Determining how these basic neuropsychological processes change across normal development and in discrete populations will inform our understanding of the neural correlates of social information processing. Finally, the Virtual School paradigm may also prove useful for delineating the neural circuits engaged by social contexts in other pediatric clinical populations, including autism spectrum and conduct disorder.

## Conclusions

SAD typically manifests in adolescence, is the most common type of anxiety disorder, and increases risk for a variety of psychiatric conditions. Much progress has been made using socially dynamic fMRI paradigms to delineate the neural circuits that promote risk for, and support expression of, SAD. However, the neural mechanisms associated with several key symptoms of SAD remain unknown. The Virtual School paradigm was developed to determine associations between brain function and behavior in specific social contexts that elicit symptoms central to SAD. We believe research based on this paradigm, and others like it, will provide a critical step toward furthering our understanding, and thus treatment of, SAD.

## Competing interests

The authors declare that they have no competing interests.

## Authors’ contributions

JMJ and EEN conceived of, and designed the Virtual School paradigm, and wrote the initial draft this manuscript. JMJ and OLW coordinated data collection and JMJ conducted statistical analyses. EL, NAF, and DSP provided critical input to the design of the Virtual School paradigm, supervised the development of software scripts needed to implement the study, and provided resources needed to acquire data. All authors contributed to, read, and approved of, the final manuscript.
